# The potential benefits of assessing post-cardiopulmonary exercise testing (CPET) in aging: a narrative review

**DOI:** 10.1186/s13102-023-00671-x

**Published:** 2023-05-01

**Authors:** Zi Xiang Lim, Bibek Gyanwali, Janjira Soh, Angela S. Koh, Jorming Goh

**Affiliations:** 1 Healthy Longevity Translational Research Program, Yong Loo Lin School of Medicine, National University of Singapore (NUS), Queenstown, Singapore; 2 Centre for Healthy Longevity, National University Health System, Singapore, Singapore; 3 Department of Biochemistry, Yong Loo Lin School of Medicine, National University of Singapore (NUS), Queenstown, Singapore; 4 National Heart Centre Singapore, Singapore, Singapore; 5 Duke-NUS Medical School, Singapore, Singapore; 6 Department of Physiology, Yong Loo Lin School of Medicine, National University of Singapore (NUS), Queenstown, Singapore

**Keywords:** Cardiopulmonary exercise testing (CPET), Cardiorespiratory, Excess post-exercise oxygen consumption (EPOC), Aerobic/anaerobic thresholds

## Abstract

Cardiopulmonary exercise testing (CPET) is an important tool to measure the cardiopulmonary fitness of an individual and has been widely used in athletic, clinical and research settings. Most CPET focus on analyzing physiological responses *during* exercise. We contend that the post-CPET recovery physiological responses offer further diagnostic and prognostic information about the health of the cardiopulmonary and metabolic systems, especially when testing apparently healthy middle-aged and older adults. However, there are limited studies that investigate physiological responses during the post-CPET recovery, and even less so in middle-aged and older adults. Therefore, this current review is aimed at discussing the contribution of post-CPET recovery parameters to cardiopulmonary health and their potential applications in aging populations. In addition to the existing methods, we propose to examine the aerobic and anaerobic recovery threshold post-CPET as novel potential diagnostic and/or prognostic tools.

## Key points

1. This current review discusses the contribution of post-cardiopulmonary exercise testing (CPET) recovery parameters to cardiopulmonary health and their potential applications in aging populations.
2. In addition to the existing methods, we propose to examine the aerobic and anaerobic recovery thresholds post-CPET as novel potential diagnostic and/or prognostic tools.

## Introduction

Cardiopulmonary exercise testing (CPET) is used to measure cardiorespiratory fitness and physiological responses to aerobic exercise in a wide variety of settings. As such, a plethora of exercise testing protocols (progressive incremental exercise to volitional exhaustion, time to exhaustion, constant load etc.) and modes (e.g. treadmill run/walk, cycling, rowing) are available to cater for different populations, ranging from athletes to geriatric and clinical populations. Often, the examined variables include maximal/peak oxygen consumption (V̇O_2_max/peak), and threshold variables (e.g. ventilatory thresholds (VT), lactate thresholds (LT) and gas exchange thresholds) [1, 2]. These threshold variables are often used to determine aerobic and anaerobic threshold of an individual, representing an individual’s submaximal cardiorespiratory fitness [1, 2].

To determine an individual’s cardiorespiratory fitness, individuals will strive to achieve his or her maximal/submaximal capacity, thereby also delineating their aerobic and anaerobic thresholds. In theory, all thresholds, in relation to either aerobic or anaerobic, should happen at the same time, for example gas exchange aerobic threshold = VT1 = LT1, but in a complex physiological system, one may happen before the other or vice versa, which depends on the individuals’ physiological system [2]. For example, the physiological system deficit in individual “A” might be the metabolic system while in individual “B” might be the pulmonary system, where both individuals may achieve similar V̇O_2_max/peak with distinct threshold points [1–5]. These distinct thresholds can thus be used for diagnosis or prognosis for specific diseases/risk factors/deficits in a complex system.

While it is certainly informative to measure cardiopulmonary fitness during CPET, the physiological state during post-exercise recovery remains an important, yet under-studied physiological parameter. The most commonly studied parameters during recovery from exercise are excess post-exercise oxygen consumption (EPOC) and heart rate recovery (HRR) [6]. The degree to which gas exchange parameters may influence post-CPET recovery have not been studied in detail. Since many physiological parameters can diagnose a risk factor or disease from exercise testing, the purpose of this review is to discuss the potential contribution of recovery parameters post-aerobic exercise (CPET and/or constant power output/speed/inclination aerobic exercise e.g. 100W cycling load for 30 min) to cardiopulmonary health, and their potential applications in middle-aged and older adults.

### The aging cardiovascular, pulmonary and skeletal muscle system

Chronological aging is associated with functional decline in the cardiovascular, pulmonary and skeletal muscle systems, particularly during dynamic, aerobic exercise. Specifically, chronological aging is associated with a gradual age-related decline in V̇O_2_max/peak as well as in the associated metabolic thresholds [7]. Cross-sectional studies suggest that the rate of decline in V̇O_2_max was faster across chronological age (males =  ~ 26.0%, females =  ~ 27.0% ml/min/kg per decade; 68 males/103 females [7]), while the decrease in ventilatory threshold (VT) (males =  ~ 13.0%, females =  ~ 13.5% ml/min/kg per decade) was less rapid with age [7]. In addition, studies suggest that the rate of decrease in V̇O_2_max accelerates after the ages of 40–50 years old, from ~ 0.3 to 0.6% per year around 20–30 years old to > 2.0% per year in 70–79 years old [8, 9]. The rate of decline in V̇O_2_max and shift in VT may be due, in part, to the age-related changes in the respiratory system [10, 11], cardiovascular system [11] and/or skeletal muscle system [12–14], given these are the major organ systems affecting an individual’s aerobic capacity [15, 16]. In addition, the rate of decline in VT being slower compared with the rate of decline in V̇O_2_max has been suggested to be due, in part, to the selective loss of type 2 skeletal muscle fibres [12–14], resulting in a relative increase in type 1 fibres [17]. The age-related decline in V̇O_2_max was also different between training status of adults in the same age group; endurance-trained adults have a higher V̇O_2_max than physically-active adults, while both groups also have higher V̇O_2_max than sedentary individuals [18]. While it is important to understand the physiological responses during CPET, analysing physiological responses during post-CPET recovery can elucidate further details of one’s cardiopulmonary/ metabolic health.

Post-CPET recovery analyses have revealed age-associated differences in cardiovascular and pulmonary variables. One study examined the effect of 70% V̇O_2_max exercise on post-exercise recovery gas exchange variables in older (~ 67.8 ± 7.5 years old; 6 males/2 females) and younger (~ 29.5 ± 6.4 years old; 16 males/6 females) adults [19]. The authors found that the recovery half-time kinetics of minute ventilation (V̇E), volume of carbon dioxide production per min (V̇CO_2_) and volume of oxygen consumption per min (V̇O_2_) during recovery was significantly slower in older adults compared with younger adults [19]. Age-related reduction in the central and/ or peripheral CO_2_ chemosensitivity contribute to the slower recovery kinetics of V̇E, V̇CO_2_ and V̇O_2_ in older adults, as there is delayed removal of exercise-induced CO_2_ production [20]. It has been postulated that age-related changes in lung mechanics and/or lung muscle strength in older adults are insufficient to explain the age-related reduction in ventilatory responses, therefore suggesting the reduced ability of chemoreceptors and mechanoreceptors to sense and generate appropriate neural and ventilatory response [21]. Therefore, more research is needed to establish the key age-related changes affecting the ventilatory responses. However, when comparing between well-trained younger (~ 24.5 ± 3.7 years old; 8 males, 4 females) and well-trained older adults (~ 47.3 ± 8.6 years old; 8 males, 4 females) there were no significant differences between post-exercise recovery kinetics [22]. These studies suggest that although aging leads to slower post-exercise recovery kinetics, exercise training status of an individual can mitigate this age-related decline which highlights the importance of maintaining regular exercise training across the lifespan.

As described above, chronological aging is associated with functional decline in the cardiovascular and pulmonary systems, observed during, and after dynamic exercise. This begs the questions as to whether individuals with age-related diseases will present with unique physiological differences during recovery from aerobic exercise. Therefore, it is important, in the context of age-related diseases, to examine physiological changes not limited to only during exercise, but also during the recovery period. Physiological measurements obtained in the post-exercise state will provide valuable insight into physiological changes during the aging process, as well as recovery from aerobic exercise in individuals with or without age-associated chronic diseases. At present, these two areas remain poorly understood. Therefore, we need to explore existing studies that have examined post-CPET variables in disease states, and how they related to the integration or malintegration of the cardiopulmonary/ metabolic systems.

### Diseased states and the determination of post-CPET variables

#### Excess post-exercise oxygen consumption (EPOC)

**Fig. 1:**
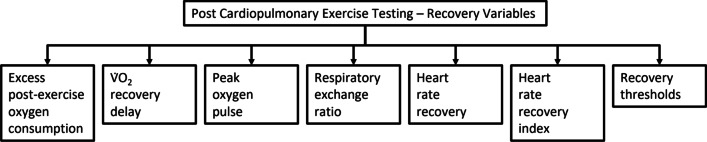
Variables that can be analyzed post-CPET, for the study of systemic function during post-exercise

Excess post-exercise oxygen consumption (EPOC) occurs after an acute bout of exercise, with an immediate increase in V̇O_2_. EPOC has been explained by the oxygen debt hypothesis, where this process is necessary for the replenishment of oxygen debt incurred to remove lactate at the onset of exercise [23–25]. EPOC has also been suggested to play a role, in part, in maintaining physiological and biochemical homeostasis, for example, by: (i) restoring adenosine triphosphate (ATP)-phosphocreatine (PCr) in skeletal muscle [26–28], (ii) replenishing blood and muscle oxygen, and (iii) redistributing ions (increased sodium–potassium pump activity) [29]. Often, EPOC is analysed for its magnitude, duration and recovery half-life. With further understanding of exercise physiology, the concept of EPOC has evolved, with the definition now stating that EPOC consists of a fast (within 1 h post-exercise) and a slow (after 1 h post-exercise) component [30]. The duration of fast and slow components are dependent on the type, intensity, and duration of exercise being performed [30]. The slow component of EPOC also supports the removal of lactate, and increased body temperature, blood flow and ventilation [30]. Hence, EPOC represents an accurate and precise means of evaluating the amount of exercise of optimal energy consumption required for health promotion [31]. Individuals with heart failure and coronary artery disease have a prolonged EPOC [32] (Fig. 1).

The EPOC half-life recovery is the time taken for V̇O_2_ to decrease from its maximal value during aerobic exercise, to 50% of V̇O_2_max during post-exercise recovery. In this context, a prolonged EPOC half-life was observed in patients with chronic heart failure (~ 97 to 155 s; 72 males; ~ 50 years old) after a bout of maximal aerobic exercise, compared with healthy individuals (~ 77 s; 13 males; ~ 50 years old) [33]. The speed of half-life recovery is related to the severity of chronic heart failure [34]. The delayed time constants of V̇O_2_ and V̇E during recovery in individuals with chronic heart failure have been explained, in part, by a delay in the recovery of energy stores in the skeletal muscle, as demonstrated by Cohen-Solal and colleagues [33]. Other factors, such as microcirculatory changes, sustained hyperpnea, carbon dioxide retention, prolonged recovery of cardiac output, and increased cost of breathing [32, 33, 35] may also contribute to the delayed EPOC in patients with chronic heart failure.

The rate at which V̇O_2_ recovers to resting values has been used, in part, as an index of systemic oxidative capacity (mainly attributed to the oxidative capacity of skeletal muscle, cardiovascular, and pulmonary) in healthy individuals [26, 33]. Thus, differences in oxygen kinetics post-CPET from individuals with age-related chronic disease can be used as a diagnostic and/or prognostic tool, for example, in individuals with chronic heart failure [32, 33] and in individuals with type 2 diabetes [36]. The differences in post-CPET oxygen kinetics can be explained, for example, by impairments in metabolic [37] and/or cardiac function [33], which can affect physiological responses post-CPET. When the analysis of post-CPET oxygen kinetics is combined with other post-CPET variables, there is potential to identify specific physiological impairments for recovery.

#### V̇O_2_ recovery delay

V̇O_2_ recovery delay is a measure of time from the end of exercise until V̇O_2_ falls permanently below V̇O_2_peak, defined and illustrated by Bailey et al. [38], where longer duration of the recovery delay represents an impaired cardiac reserve capacity in individuals with heart failure. Heart failure patients with preserved ejection fraction (64 ± 10 years old; 15 males, 15 females), or with reduced ejection fraction (62 ± 11 years old; 18 males, 2 females) had prolonged V̇O_2_ recovery delays, compared with healthy control (58 ± 13 years old; 13 males, 9 females) participants (25 s vs. 28 s vs. 5 s, respectively) [38]. Further, V̇O_2_ recovery delay was inversely related to cardiac output augmentation during exercise in both types of heart failure patients [38]. Therefore, measuring V̇O_2_ recovery delay could be used to assess cardiac reserve capacity [38]. Future work should also determine whether V̇O_2_ recovery delay is associated with other age-related conditions, such as peripheral vascular disease, and whether it has prognostic potential.

#### Peak oxygen pulse

Peak oxygen pulse is calculated by dividing V̇O_2_max by maximal heart rate (HR) during exercise and is an indicator of stroke volume and arteriovenous oxygen (a-vO_2_) difference when corrected for lean body mass [39]. In cardiac patients with mild-to-moderate heart failure (HF) followed for 19 ± 12 months, those without major cardiac events had higher absolute (11.4 vs. 9.2 ml/beat) and body fat-adjusted peak oxygen pulse (15.6 vs. 11.9 ml/beat), compared with patients that suffered major cardiac events [39]. Low peak oxygen pulse was the strongest predictor of clinical events (chi sq 10.5), independent of body fat, while peak oxygen pulse adjusted for body fat showed an even stronger prediction (chi sq 12.4) [39]. In the same study, even in most subgroups (including women, obese subjects, those receiving beta-blockers, and those with class III HF), peak O_2_ pulse normalized for lean mass was similar to, or superior to peak VO_2_ for predicting major clinical events [39]. Therefore, oxygen pulse during recovery from CPET offers an attractive means to uncover potential pathophysiological outcomes in the cardiopulmonary system.

#### Respiratory exchange ratio (RER)

Respiratory exchange ratio (RER), CO_2_ produced/O_2_ consumed, is an indirect measure of skeletal muscle capacity for oxidative phosphorylation [40]. Post-exercise RER can be used to measure the RER overshoot- defining the highest RER value during exercise (A) and recovery (B), and defining the percentage magnitude between these 2 points (A) and (B) [41]. Time to RER max was defined by duration between (A) and (B) [41]. Individuals with kidney transplant (51.4 ± 13.0 years old) showed significant RER overshoot, with a RER magnitude lower than healthy individuals; further, the RER magnitude was able to stratify according to fitness levels [41]. This could be due to the reductions in capillary density, mitochondria density and/or the increased diffusion distance within skeletal muscle of individuals with kidney failures [42].

#### Heart rate recovery (HRR)

Heart rate recovery (HRR) has been suggested to reflect the balance between the reactivation of the parasympathetic nervous system and the withdrawal of the sympathetic nervous system- a delayed HRR would suggest a potential ailment in these systems. Indeed, parasympathetic nervous system reactivation is the main contributor to HRR differences obtained post-CPET, as observed between athletes (20 ± 2 years old) and patients with chronic heart failure (55 ± 12 years old) but not with healthy, but sedentary age-matched young (20 ± 4 years old) and old adults (56 ± 6 years old), with the differences being most significant at 30 s post-CPET [43].

As well, HRR has been used to predict the onset of coronary heart disease [44], cardiovascular-related mortality [45], non-cardiovascular mortality [46], and all-cause mortality [44, 47]. Up to one minute post-CPET HRR can predict mortality, with HRR at 10 s after CPET being the greatest predictor [48]. In addition, HRR does not coincide with the return of V̇O_2_, V̇CO_2_ and V̇E to pre-exercise levels, particularly in older adults [49], suggesting that HRR is an independent marker of aging.

#### Heart rate recovery index (HRRI)

Heart rate recovery index (HRRI) is another measure of HRR and is defined as the ratio of acceleration time of HR during exercise (time from baseline HR to HR max) to the deceleration time of HR (time from HR max to baseline HR) post-CPET [50]. Cozlac and colleagues [50] assessed whether HRRI can be used to predict the response of cardiac patients to cardiac resynchronization therapy, a therapy to normalize patients’ heart rhythm. They found that responders and non-responders to cardiac resynchronization therapy had significant differences in HRRI post-CPET [50]. The differences were associated with the cardiac phenotype and function, where responders to cardiac resynchronization therapy had significant left ventricular reverse modelling and larger left ventricular ejection fraction as compared to non-responders [50].

HRRI has also been used to assess aerobic fitness in a healthy cohort, where HRRI can predict V̇O_2_max, and maximum speed during CPET at 1 min post-exercise, and up to 2 and 3 min post-exercise for females (n = 130; average age = 24.6 years old), and males respectively (n = 718; average age = 27.6 years old) [51]. Future studies can examine the potential for HRRI post-CPET in middle-aged and older adults.

### Prognostics for healthy aging

Given that the above recovery variables can be used to diagnose individuals with diseases, the next section will discuss the potential use of these recovery variables as a prognostics tool for healthy aging.

#### EPOC

In older adults (67.8 ± 7.5 years old), the slower recovery kinetics of V̇E, V̇CO_2_ and V̇O_2_, as compared to younger (29.5 ± 6.4 years old) adults [19], were attributed partly to age-related decreases in central and/or peripheral CO_2_ chemosensitivity, which delay the removal of exercise-induced CO_2_ [20] In addition, for functionally impaired older adults, EPOC was a better predictor for functional performance than either VO_2_peak or VO_2_ during exercise [52], compared with age-matched, functionally competent adults, whose VO_2_ values during exercise testing were better predictors for functional performance [52].

In another study, oxygen uptake in physically inactive, middle-aged adults during aerobic exercise was similar between continuous cycling (30 min at 60% V̇O_2_max) or interval cycling (alternating bouts of 80% V̇O_2_max (2 min) and 40% V̇O_2_max (1 min) repeated 6 times) [53]. However, interval cycling resulted in significantly higher EPOC, compared with continuous cycling, despite similar energy expenditure during both exercise modalities [53]. One explanation could be due to the energy demands between the two exercise modalities, with interval exercise more reliant on anaerobic glycolysis, compared with continuous cycling. Thus, there is greater production of H^+^ ions, decreased efficiency of recovery of metabolic pathways, thereby increasing oxygen demand post-exercise (observed as EPOC) during recovery.

#### RER

The training status of an individual indicates exercise tolerance. Frey and colleagues examined RER during post-exercise recovery from low- (LI; ~ 65% V̇O_2_max) versus high-intensity (HI; ~ 80% V̇O_2_max) aerobic exercise, between trained (27.8 ± 2.6 years old; 6 females) and untrained individuals (24.3 ± 1.6 years old; 7 females), and found no significant differences between groups [54]. However, HI exercise resulted in higher RER compared with LI exercise in both groups [54]. Furthermore, RER decreased rapidly within the first 10 min following LI and HI, and was lower than baseline throughout 60 min of recovery in untrained individuals [54]. In the trained individuals, RER also decreased rapidly through the first 10 min after LI exercise, and 20 min after HI exercise [54]. The lower RER observed during post-exercise in untrained participants suggests that untrained individuals relied more on fat oxidation during post-exercise recovery, compared with trained individuals. Also, lower RER during the post-exercise period may implicate CO_2_ retention in bicarbonate pools, leading to a state of acidosis by increasing hydrogen ions and lowering of pH.

#### Advantages of measuring post-CPET variables

Although some recovery kinetics are associated with cardiorespiratory fitness variables measured during exercise, the recovery oxygen kinetics has advantages of being independent of the level of exercise [32, 33, 55], allowing its use as a prognostics biomarker when individuals are unable to achieve maximal efforts during exercise. In addition, V̇O_2_peak determination depends on each individual’s motivation and test termination criteria, where both do not influence the oxygen kinetics of recovery.

Indeed, recovery oxygen kinetics is able to further prognose individuals with heart failure, where lower recovery V̇O_2_ is associated with higher mortality, apart from using only oxygen kinetics during CPET [56]. In addition, recovery V̇O_2_ is a better predictor than V̇O_2_peak at mortality of individuals with cardiac heart failure [56].

Combined post-CPET variables such as gas exchange and heart rate have been observed to differentiate three different forms of congenital heart lesions [57]. Although this example is not an age-related disease, it provides insights into how post-CPET variables can indicate impairment in human physiology. More studies are necessary to establish how post-CPET variables are related to the physiological systems.

Given the limited studies available for post-CPET variables on the aging population, more research is needed to further understand whether recovery kinetics can prognose other age-related diseases and improve understanding of aging physiology.

### Concept of recovery variables analysis (absolute values vs. thresholds)

**Fig. 2:**
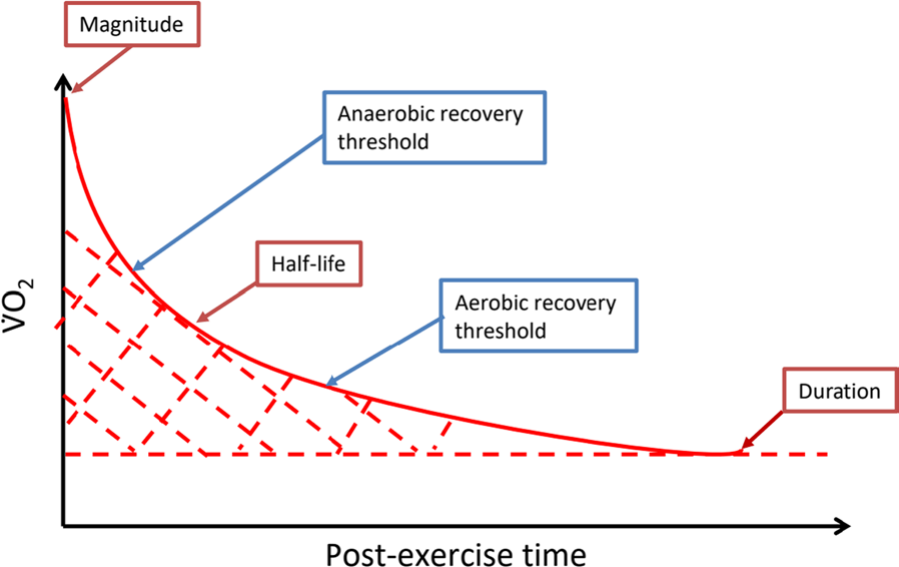
An example of a typical EPOC absolute measurements (red boxes and arrows: magnitude, duration, and area under curve) and the proposed recovery threshold measurements (blue boxes and arrows: anaerobic and aerobic recovery thresholds)

By further establishing which other gas exchange variables is able to indicate health risk factors, exercise interventions can be explored and recommended to prevent or minimise the potential age-related pathologies. However, most studies have analysed absolute points on individual gas exchange variables. For example, EPOC is commonly analysed for its magnitude (highest point), duration (back to baseline), and half-life (mid-point) (Fig. 2), similar to other variables measured during recovery such as HRR. These recovery variables have not been observed from a recovery threshold perspective (aerobic and anaerobic recovery thresholds; Fig. 2), where V̇O_2_ and V̇CO_2_ can be plotted to determine the point where recovery aerobic threshold occurs (inflection during recovery). Based on the concept where the 2 thresholds (aerobic and anaerobic) occur, we propose in this paper, for future studies to examine recovery thresholds in the healthy and diseased population.

Applying the concept of determining an individual’s aerobic threshold rather than absolute V̇O_2_peak [2], here we propose the measurement of recovery threshold during post-CPET, apart from the absolute points of an individual variable such as EPOC.

### Recovery threshold measurement

**Fig. 3:**
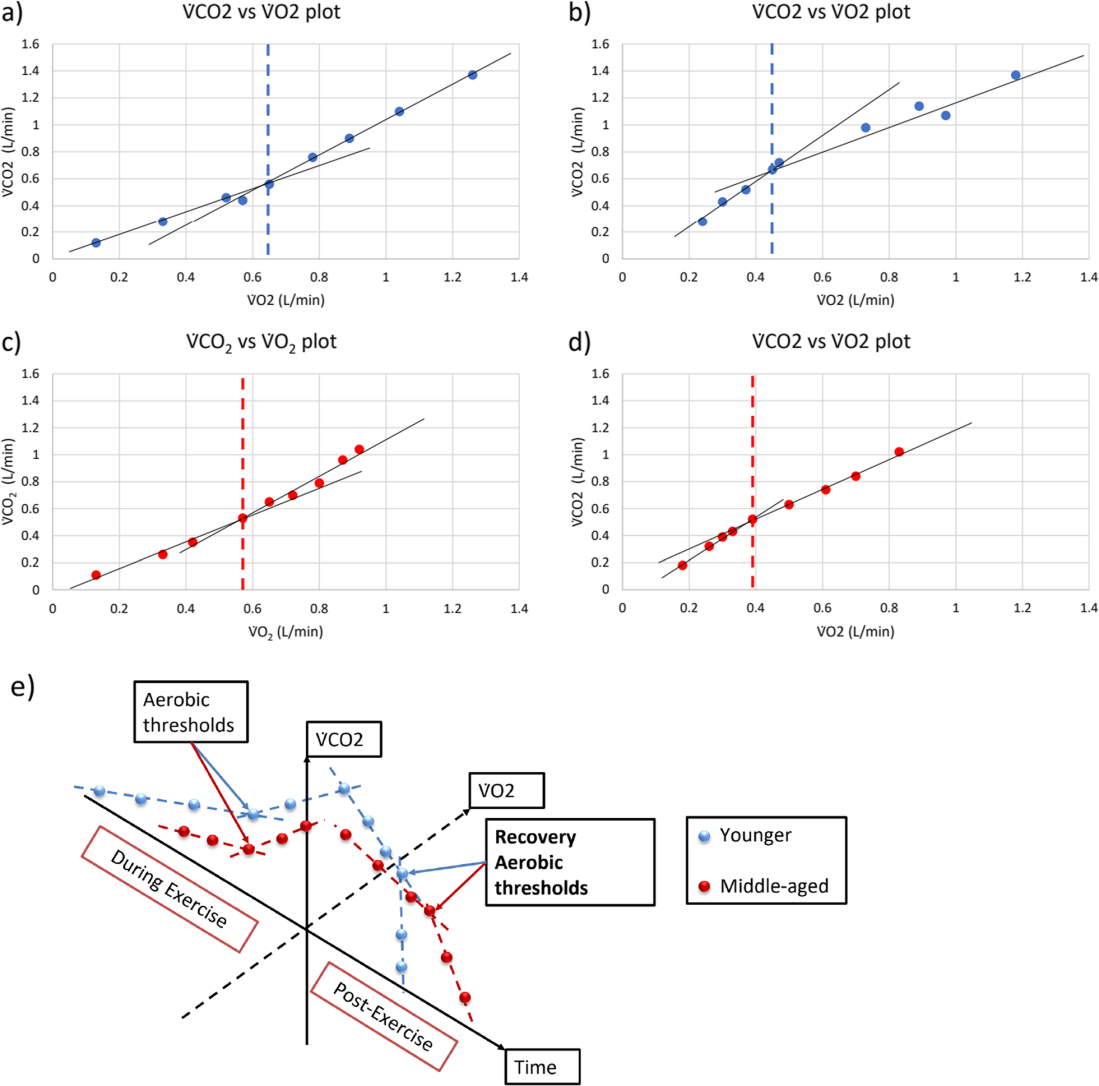
Schematic illustration plots of ˙V̇CO_2_ against V̇O_2_ **a** during exercise and **b** post-exercise of a younger female adult, and **c** during exercise and **d** post-exercise of a middle-aged female adult **e** hypothetical 3-dimensional graph across time (during CPET and post-CPET), comparing one younger and one middle-aged adults, where a to d are expressed in a 3-dimensional graph across time (illustration not drawn to scale). Dotted lines represent aerobic threshold of a younger (**a**; blue dotted line) and a middle-aged adult (**c**; red dotted line). Proposed recovery aerobic threshold of a younger (**b**; blue dotted line) and a middle-aged female adult (**d**; red dotted line) (unpublished results)

Traditionally, to detect the aerobic threshold during a bout of exercise, one would examine two variables against each other, for example, plotting V̇CO_2_ against V̇O_2_; the inflection point would then indicate the aerobic threshold (Fig. 3a), or plot V̇E against V̇CO_2_; the inflection point would indicate anaerobic threshold. From a recovery perspective, these recovery thresholds have not been explored and we speculate whether the inflection point (Fig. 3b) could suggest the recovery of aerobic or anaerobic threshold systems (Fig. 2). These recovery thresholds (aerobic or anaerobic systems) can indicate the demands on the cardiopulmonary/metabolic systems to return to baseline and to provide prognostic value for identifying age-related physiological decline. In addition, the slope of the recovery variable of interest, for example, the initial slope prior to the recovery threshold, and the slope after the recovery threshold (Fig. 3b) can be used to identify age-related physiological deficits, where they can be compared across age-related conditions (Fig. 3c and d). In addition, when Fig. 3a–d are combined together in a proposed 3-dimensional model (Fig. 3e), the area under the 3-dimensional curve can be compared (Fig. 3e). In addition, this concept can be applied to the proposed anaerobic recovery threshold, by using the V̇E against V̇CO_2_ graph for example. Of course, future studies are required to examine this potentially novel method of measuring recovery aerobic and anaerobic thresholds.

Given that populations with age-related diseases such as individuals with type 2 diabetes, having metabolic impairment such as lower capacity to utilise carbohydrate [58] and individuals with heart failure with impaired cardiac reserve capacity has been observed with a delayed V̇O_2_ recovery [38], these populations may potentially have a slower recovery threshold with a larger area under the curve for post-exercise, compared with the healthy population.

## Conclusions

The use of CPET has mostly been explored during the exercise phase, with limited number of studies reporting the post-exercise recovery phase. Typically, variables (EPOC, HRR, RER) that were examined during post-exercise recovery phase were individually analysed. In this narrative review, we have proposed the idea of analysing the post-exercise recovery relationship between variables, such as plotting V̇CO_2_ against V̇O_2_, for the analysis of recovery thresholds, and the time taken to achieve these thresholds post-exercise. The analysis of post-CPET variables could potentially provide further understanding of aerobic and anaerobic recovery thresholds, and the potential to have significant utility in age-associated disease prognostication.

## Data Availability

Not applicable.

## Abbreviations

EPOC: Excess post-exercise oxygen consumption
HR: Heart rate
HRR: Heart rate recovery
HI: High intensity
L: Liters
LI: Low intensity
LT: Lactate Threshold
V̇O_2_max/peak: Maximal/Peak oxygen consumption
V̇E: Minute ventilation
min: Minute
RER: Respiratory exchange ratio
VT: Ventilatory threshold
V̇CO_2_: Volume of carbon dioxide produced per min
V̇O_2_: Volume of oxygen consumed per min
